# Omega-3 Hastens and Omega-6 Delays the Progression of Neuropathology in a Murine Model of Familial ALS

**DOI:** 10.2174/1874205X01711010084

**Published:** 2017-12-22

**Authors:** Edward F. Boumil, Rishel Brenna Vohnoutka, Yuguan Liu, Sangmook Lee, Thomas B Shea

**Affiliations:** 1 Laboratory for Neuroscience, University of Massachusetts Lowell, Lowell, MA 01854, USA; 2 Department of Biomedical and Nutritional Sciences, University of Massachusetts Lowell, Lowell, MA 01854, USA

**Keywords:** Omega-3, Omega-6, Neuropathology, Amyotrophic lateral sclerosis, Motor neuron, Inflammation

## Abstract

**Background:**

Amyotrophic lateral sclerosis (ALS) is a progressive disease of motor neurons that has no cure or effective treatment. Any approach that could sustain minor motor function during terminal stages would improve quality of life.

**Objective:**

We examined the impact of omega-3 (Ω-3) and Ω-6, on motor neuron function in mice expressing mutant human superoxide dismutase-1 (SOD-1), which dominantly confers familial ALS and induces a similar sequence of motor neuron decline and eventual death when expressed in mice.

**Method:**

Mice received standard diets supplemented with equivalent amounts of Ω-3 and Ω-6 or a 10x increase in Ω-6 with no change in Ω-3 commencing at 4 weeks of age. Motor function and biochemical/histological parameters were assayed by standard methodologies.

**Results:**

Supplementation with equivalent Ω-3 and Ω-6 hastened motor neuron pathology and death, while 10x Ω-6 with no change in Ω-3 significantly delayed motor neuron pathology, including preservation of minor motor neuron function during the terminal stage.

**Conclusion:**

In the absence of a cure or treatment, affected individuals may resort to popular nutritional supplements such as Ω-3 as a form of “self-medication”. However, our findings and those of other laboratories indicate that such an approach could be harmful. Our findings suggest that a critical balance of Ω-6 and Ω-3 may temporarily preserve motor neuron function during the terminal stages of ALS, which could provide a substantial improvement in quality of life for affected individuals and their caregivers.

## 1 INTRODUCTION

Amyotrophic Lateral Sclerosis (ALS) is characterized by a progressive loss of motor neurons, with eventual degeneration of muscles themselves, resulting in paralysis and death. There currently is no cure or long-lasting treatment. Mutations in multiple genes underlie ALS, including Cu/Zn superoxide dismutase (SOD1) [1-4]. SOD1 is abundant within motor neurons, where it converts superoxide anions to hydrogen peroxide. More than 90 different mutations have been described that span all exons. SOD1 mutations promote neurodegeneration by a gain of toxic function, which promotes increased reactive oxygen species and widespread oxidative damage to proteins, nucleic acids and lipids. Importantly, however, mutations in SOD-1 account for only 20% of the familial cases, which themselves make up only 5-10% of the total cases. Clearly, one or more additional factors is/are involved in the onset and/or progression. Such factors remain elusive despite decades of study [2, 3]. Several genes have been implicated in ALS to date, such as C9ORF72 and TARDBP [4].

ALS is accompanied by inflammation, mediated in part by astrocyte and microglial activation [5, 6]. Proinflammatory activity of cyclooxygenases COX-1 and 2 is upregulated in ALS; suppressing this activity, and/or activation of glia, to reduce inflammation represents a therapeutic target [5-7]. COX-1 and 2 convert dietary polyunsaturated fats (PUFAs) into a series of thromboxanes and prostaglandins; those derived from Ω-6 are proinflammatory, while those derived from Ω-3 are anti-inflammatory [8, 9]. These collective findings prompted the consideration that Ω-3 would be beneficial during ALS pathology. Accordingly, Yip and colleagues supplemented SOD-1 mice with 1000x Ω-3. This 1000x increase corresponds to common over-the-counter Ω-3 supplements for human consumption. While 1000x Ω-3 suppressed inflammation in SOD-1 mice, it unexpectedly hastened the progression of motor neuron disease [10]. While this seems to contradict the recent clinical study reporting a beneficial effect of consuming Ω-3, we note that participants in that study also consumed similar amounts of Ω-6 [11]. We therefore considered that clinical benefit may have been achieved by supplementation with a balance of Ω-3 and Ω-6 rather than Ω-3 alone. As shown herein, supplementation of SOD-1 mice with Ω-3 along with similar or increased levels of Ω-6 delayed the progression of motor neuron disease.

## 2 MATERIALS AND METHODS

All procedures were approved by our Institutional Animal Care and Use Committee.

### 2.1 Mice and Diets

B6SJL-TgN(SOD1-G93A)1Gur mice (hemizygotes; Jax stock #JR2726), which express the Gly93>Ala substitution of human SOD1 were maintained on (1) a standard mouse diet (where mice consumed <1mg/kg/day of Ω-3 and Ω-6); (2) this diet supplemented with Ω-3 and Ω-6 such that mice would consume approx. 4mg/kg/day of each (“4xΩ-3,6 diet”), or (3) supplemented with Ω-6 such that mice would consume approx. 11mg/kg Ω-6 while maintaining Ω-3 at <1mg/kg/day (“10xΩ-6 diet”) commencing at 4 weeks of age (prior to onset of motor neuron decline) [12, 13]. Diets were prepared by Test Diets (Purina). The total energy derived from polyunsaturated fatty acids (PUFAs) was the same in the 4xΩ-3,6 and 10xΩ-6 diets (approx. 23%). These diets were designed to approximate PUFA ratios in the so-called Mediterranean and Western diets, respectively [14].

### 2.2 Motor Neuron Function

Motor function was monitored in 3 groups of 3 mice each commencing at 8 weeks of age using classical approaches. “Disease Progression” is classically defined as the inability of a mouse to right itself within 10 sec when placed on its back, at which point the mouse is euthanized for humane reasons. This time is defined as “surrogate death” and scored with equal weight as actual death [12, 13, 15]. Disease Progression therefore refers to “progression to death,” and allows additional determinations such as the initial loss of mice and the 50% survival point. “Disease Onset” is classically defined as a sharp decline in the length of time that a mouse can cling to a wire by its front paws when suspended above its cage bedding. The maximum time for this test is 1min, which is readily achieved by normal mice and by SOD-1 mutant mice prior to motor neuron decline. Disease onset occurred at 15 weeks of age in SOD-1 mice without intervention [12, 13]. Additional tests that quantify more subtle aspects of motor function included (1) Motor Activity, scored as 100% for mice with no detectable deficits, 75% for abnormal gait with hind-limb tremors, 50% for bilateral hind-limb paresis but mobile, 25% for bilateral hind-limb paralysis and 0% for death [12, 15]; (2) the Quadrant test, where the frequency of mice crossing into a new cage quadrant is recorded over 30sec, and (3) an overall Activity assay, where mice received 1 point each for moving around the cage, digging into bedding, jumping, and reaching up along the cage wall (or a 0 for lack of each of these activities) over a 5min interval. Tests were initially performed weekly and daily once any decline was observed. Performance of each mouse was calculated as a percentage of that exhibited at its own baseline. Mean performance (± standard error) was then be determined for all mice on each diet. Statistical comparison among all groups was carried out by ANOVA with Fisher’s post-hoc determination. To compare our findings with those of Yip **et al.** [10], who provided mice with a diet supplemented with 1000x- Ω-3 with no change in Ω-6, we included their findings on survival of mice maintained on this diet in graphing our survival curves. To accomplish this, we normalized respective 50% survival points on the standard diet, then adjusted Ω-supplemented diets by the same scale.

### 2.3 Quantification of Motor Neurons and Glia

Mice were sacrificed after 5 and 6 weeks on the above diets by cervical dislocation. Spinal cords were rapidly removed; a portion (40-50mg) of the thoratic spine was immediately frozen in liquid nitrogen, and the lumbar portion immersed in 4% paraformaldehyde. Motor neurons, classically identified by their large SMI-32-positive perikarya (≥20µM) and prominent nuclei and nucleoli in corresponding phase-contrast images, were quantified within paraffin-embedded sections of lumbar spinal cord. Reactive astroglia (readily identified by their intense GFAP-positive, stellate appearance) [12, 16] were quantified by co-staining of sections with anti-GFAP. Additional sections were quantified for RCA-1 levels as an index of microglial activation [17].

### 2.4 Analyses of Metabolites 

Prostaglandin and thromboxane metabolites derived from Ω-3 and Ω-6 were quantified by liquid chromatographic mass-spectrometry (LC-MS) of lumbar spinal tissue *via* standard methodologies, with identification by co-migration of purified standards as described [18].

## 3 RESULTS

**Fig. (1):**
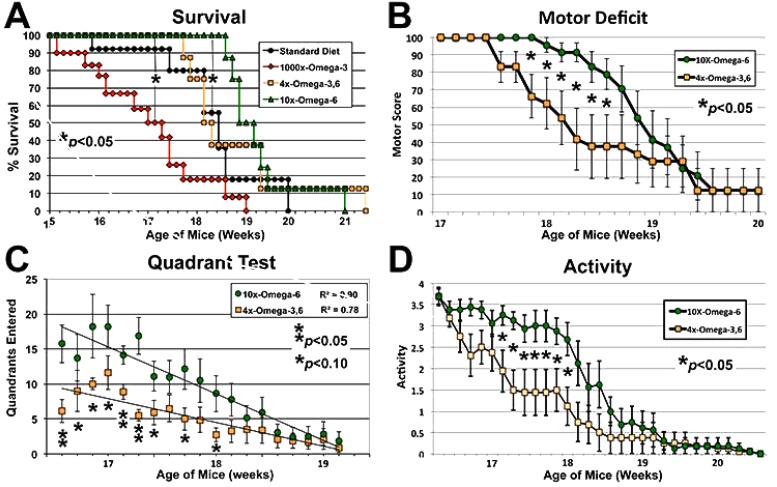
Ω-6 delayed motor neuron decline. Mice maintained on the indicated diets commencing at 4 weeks of age were subjected to motor function assays at the indicated ages. The 1000xΩ-3,6 diet (Yip *et al.*, 2013) hastened, while the 10xΩ-6 diet delayed, the 50% Survival point (panel C; p<0.05 for both, ANOVA asterisks) as compared to the standard diet (with no supplementation with either Ω). The 10xΩ-6 diet significantly (p<0.05; Student’s t tests; asterisks) sustained motor function for 1-1.5 weeks longer than the 4xΩ-3,6 as assayed by Motor Deficit (panel B), Quadrant test (panel C, trend lines calculated via Excel) and overall Activity (panel D).

Disease Onset as quantified by the standard “wire cling” test was not altered by supplementation (not shown); however, 1000x Ω-3 significantly decreased survival: when the 50% survival point was reached for this diet, 90-100% of mice on the other diets were still alive (Fig. **1A**). By contrast, 10xΩ-6 significantly prolonged survival; 100% of mice on this diet were still alive when only 50% of mice on the standard and 4xΩ-3,6 diets survived and 100% mice on the 1000xΩ-3 diet had died (Fig. **1A**). The 4xΩ-3,6 diet did not statistically alter survival vs. the standard diet. Death was first observed with the standard diet at 15.8 weeks of age, but not until 18.7 weeks of age with 10xΩ-6 (Fig. **1A**), which represents an 18% increase in survival time. Motor neuron function was also preserved for 1-2 weeks longer with 10xΩ-6 (Fig. **1B**-**D**).

**Fig. (2):**
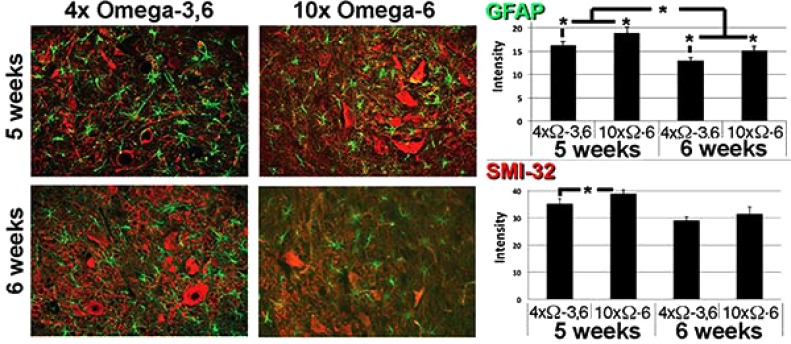
Ω-3 and Ω-6 altered reactive astrocytes and motor neuron number Representative sections of lumbar spinal cord of mice receiving the indicated diets for 1 week. Sections were reacted with anti-GFAP and SMI-32 (to reveal reactive astrocytes and motor neurons, respectively). The accompanying graphs present quantification of immunoreactivity from 10 sections from multiple cords. *p<0.05.

**Fig. (3):**
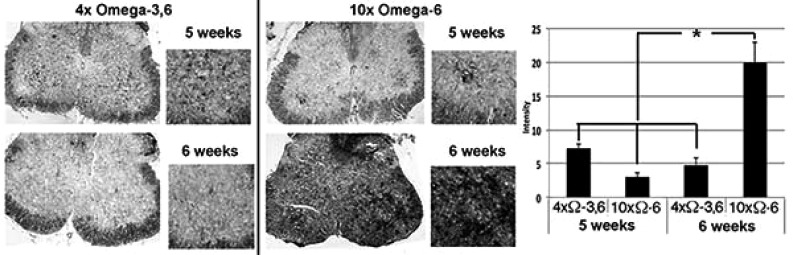
Ω-3 and Ω-6 altered microglial activation.

Motor neuron numbers, reactive astrocytes and activated microglia were quantified at 5 and 6 weeks of age (corresponding to 1 and 2 weeks of supplementation and prior to the onset of terminal symptoms; *e.g.*, Fig. (**1**). Motor neuron numbers and reactive astrocytes decline during this pre-symptomatic interval [12, 13]. However, we observed significantly greater numbers of motor neurons, reactive astrocytes and activated microglia in mice receiving 10xΩ-6 vs. 4xΩ-3,6 (Figs. **2**, **3**).

**Fig. (4):**
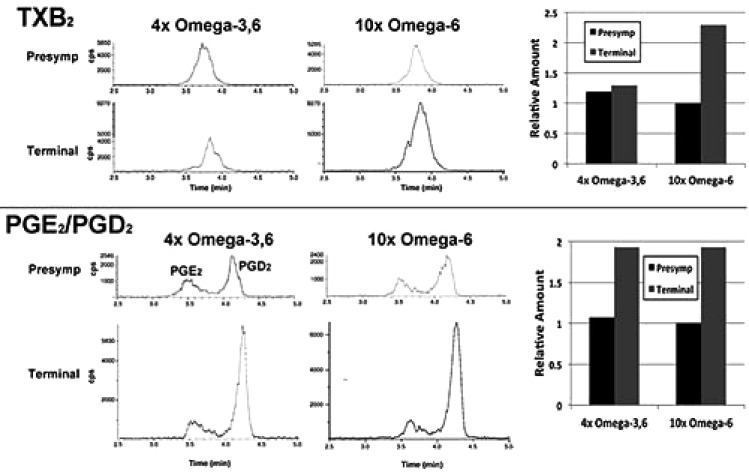
LC-MS analyses of TXB_2_, PGD_2_ and PGE_2_ standards and in homogenates of lumbar cord harvested after 1 week on indicated diets (“pre-symptomatic”) or following sacrifice at terminal stage (“Terminal). The accompanying graphs present the area under the curves to facilitate comparison. All three compounds increased >2-fold for mice maintained on the 10xΩ-6 diet; TXB_2_ did not increase for mice maintained on the 4xΩ-3,6 diet.

In a single preliminary experiment, supplementation with 10xΩa-6 induced a >2-fold increases in thromboxane 2 (TXB_2_) and prostaglandins D_2_ and E_2_ (PGD_2_/ PGE_2_) during terminal stages; 4xΩ-3,6 induced the same increase in PGD_2_/PGE_2_ but completely prevented the increase in TXB_2_ (Fig. **4**).

## 4 DISCUSSION

We report the unexpected result that dietary supplementation with equivalent Ω-3 and Ω-6 hastened motor neuron pathology and death, while 10x Ω-6 with no change in Ω-3 significantly delayed motor neuron pathology, including preservation of minor motor neuron function during the terminal stage of a mouse model of familial ALS. These findings support and extend the studies of Yip **et al.** [10], in which supplementation with Ω-3 hastened a decline in motor neuron function, by demonstrating a dose-dependent delay of ALS-related neuropathology following moderate increases in Ω-6. Acceleration of disease progression with increasing Ω-3, yet delay of disease progression with increasing Ω-6 supports the hypothesis that a balance of Ω-3 and Ω-6 may be beneficial for ALS. While these results in a mouse model seems to contradict the recent clinical study reporting a beneficial effect of consuming Ω-3, we note that participants in that study also consumed similar amounts of Ω-6 [11]. The possibility therefore remains that clinical benefit was actually achieved by supplementation with a balance of Ω-3 and Ω-6 rather than Ω-3 alone.

We observed the intriguing preliminary result that TXB_2_ generation was suppressed by the 4xΩ-3,6 diet. Since TXB_2_ is derived exclusively from Ω-6 in the nervous system [8], it is possible that the anti-inflammatory properties of Ω-3 suppressed TXB_2_ generation. Moreover, since microglia are the source of TXB_2_ in the nervous system [19], attenuation of microglial activation is a likely mechanism by which Ω-3 supplementation prevented the TXB_2_ increase Fig. (**3**). Further studies are required to substantiate these possibilities. For example, it remains to be determined whether further increase in Ω-3 suppresses additional Ω-6 inflammatory compounds (*e.g.*, PGD_2_/PGE_2_); this may have fostered the deleterious impact of 1000x Ω-3 with no compensatory increase in Ω-6 [10]. The clinical implication of this finding is supported by the unexpected observation of downstream benefits in motor neurons in a murine ALS model following supplementation with prostaglandins D2 and PEGs, which are downstream products generated from Ω-6 by COX-2 [20, 21]. These results encompass the challenge that any and all inflammation, including that resulting from consumption of Ω-6, is *de facto* deleterious. Inappropriate levels of Ω-6 are indeed associated with increased risk of cardiovascular disease, cancer and inflammatory disorders, while appropriate levels of Ω-3 reduces these risks [14]. However, several studies support establishment of an appropriate ratio of these PUFAs, rather than elimination of Ω-6: *e.g.*, (1) a ratio of 4:1 Ω-6:Ω-3 decreased mortality in secondary prevention of cardiovascular disease by 70%; (2) a ratio of 2.5:1 reduced colorectal and breast cancer proliferation, but 4:1 did not; (3) 2-3:1 suppressed rheumatoid arthritic inflammation; (4) 5:1 was beneficial for asthma, but 10:1 was deleterious. The optimal ratio is disease-specific and likely varies in accord with an individual’s genetic predisposition. In this regard, the potential benefit of astroglial and microglial activation (which accompany inflammation) has been advanced in neurodegenerative conditions, including ALS [5, 22]. Comparison of the impact of our omega-supplemented diets on these parameters in wild-type mice would be of interest.

Our findings hold the promise that a simple, non-invasive approach could be initiated presymptomatically in familial ALS (since individuals are likely aware of the possibility of inheritance and could be tested for presence of the conferring mutation), as well as administered late, even *via* a feeding tube (which routinely accompanies end-stage ALS). Notably, we initiated dietary supplementation prior to onset of symptoms, and we therefore have no indication as to whether or not supplementation would be beneficial after symptomatic manifestation; this will be examined in future studies. Cyclooxygenases (COX-1 and 2) are upregulated in ALS. These enzymes convert dietary PUFAs into thromboxanes and prostaglandins; those derived from Ω-6 are inflammatory while those from Ω-3 are anti-inflammatory. Since Ω-6 and Ω-3 compete for these enzymes, simple dietary manipulation provides a means of regulation of inflammatory pathways without attempting direct enzymatic inhibition; dietary manipulation is therefore ideal for our proposed exploratory clinical studies. Development of precise temporal and spatial promotion of controlled inflammation by pharmacological methodologies may represent an important facet of therapeutic intervention.

## CONCLUSION

ALS has no cure or effective treatment. Our demonstration of temporary preservation of motor function in mice relates directly to the quality of life for affected humans: the progressive decline in human motor function eventually prevents speech, the minor finger movement that could operate a computer (with speech capabilities), swallowing and, ultimately, breathing. The terminal stage, (complete loss of movement but prior to death) leaves the individual in a so-called “locked in” state that can last for a year or more. Any approach that could temporarily preserve minor motor function (*e.g.*, finger movement) during this terminal stage would vastly improve an individual’s quality of life, and that of caregivers, by allowing continued communication. Our findings hold the promise that a critical balance of Ω-3 and Ω-6 could provide this temporary window: mice live for 2.5-3yrs; the 1.5 to 3-week increase in survival demonstrated herein extrapolates to ≥1yr of human life based on an 80yr human life span. Moreover, we observed a window of preserved minor motor function during this period. Equally as important, affected individuals may resort to nutritional supplements as a form of self-medication [23]; based on the above preclinical findings, coupled with the popularity of Ω-3, such an approach could actually be harmful.
